# Bad for Girls and Boys: Gender Does Not Modify the Negative Effect of Physical Inactivity on Life Satisfaction in Adolescents

**DOI:** 10.3389/fpubh.2022.904411

**Published:** 2022-07-12

**Authors:** Zsuzsa Lábiscsák-Erdélyi, Annamária Somhegyi, Ilona Veres-Balajti, Karolina Kósa

**Affiliations:** ^1^Department of Physiotherapy, Faculty of Public Health, University of Debrecen, Debrecen, Hungary; ^2^National Center for Spinal Disorders, Budapest, Hungary; ^3^Department of Behavioural Sciences, Faculty of Public Health, University of Debrecen, Debrecen, Hungary

**Keywords:** life satisfaction, physical inactivity, gender as modifier, school health promotion, health promotion

## Abstract

**Objectives:**

Physical activity (PA) has a positive effect on life satisfaction (LS) among adolescents, but the moderating effect of gender and level of PA intensity have been equivocal. Our aim was to examine the pattern of physical activity by grade in high school students, and the role of gender and grade on the association between physical activity and life satisfaction.

**Methods:**

Four repeated cross-sectional online questionnaire surveys between 2011 and 2013 were carried out among all students in one Hungarian high school (*N* = 3,450). Health status and behavior was assessed by the Hungarian online version of the health behavior of school-aged children (HBSC) questionnaire. Regression with robust variance estimator was used to identify determinants of life satisfaction.

**Results:**

Good self-reported health as opposed to bad increased life satisfaction by 0.30 standard deviation; having very well or well-off family as opposed to not well-off increased LS by 0.16 standard deviation; and being inactive compared to being vigorously active decreased LS by 0.1 standard deviation.

**Conclusions:**

Physical inactivity has a negative effect on life satisfaction in boys and girls regardless of grade but compounded by low perceived family wealth.

## Introduction

Subjective wellbeing has been a topic of increasing interest in the past half century due to the globally increasing value of the individual, the importance of subjective aspects of evaluating life, and the increasing understanding that human development extends way beyond economic prosperity (1, 2). Life satisfaction (LS) is one important component of subjective wellbeing that captures a subjective evaluation of overall quality of life (1), most frequently measured by the single-item Cantril Self-Anchoring Striving Scale (3) [or Cantril ladder, not to be confused with the Satisfaction with Life Index (4)]. Research on LS has been dominated up to a decade ago by a focus on adults with majority of studies carried out in North America (5). The largest repeated cross-national survey on adolescents, the Health Behavior in School-aged Children (HBSC), introduced life satisfaction as a mandatory variable in 2001/2002 (6). LS among adolescents has been found to be related to health status across age and gender (7) and has been associated with social relations (8), socioeconomic status (9), and a range of health behaviors, including physical activity (PA) (10). However, the level of intensity of PA associated with positive effect on life satisfaction (11), as well as gender being a moderating variable for PA on LS have been equivocal (12, 13). The relationship between poor physical health and perceived life satisfaction was described by Zullig et al. (14) in 2005. Piko et al. (15) had also found that physically more active students not only have a better self-perceived health and higher levels of life satisfaction but also lower levels of depressive symptoms and less extrinsic values as life goals for their future. Self-esteem was found to be a more important predictor of LS than PA, and it also modified their relationship. However, life satisfaction in this study was assessed by the 3-item “Students' Life Satisfaction Scale,” and not by the Cantril ladder (16). High level of physical activity compared to low level was significantly associated with reduced odds of low self-esteem and low life satisfaction among senior high school students in a study by Guddal et al. (17); moreover, high level of PA was also significantly associated with reduced odds of psychological distress.

Our aim was to examine the pattern of physical activity by grade in high school students and the role of gender on the relationship between physical activity and life satisfaction in a pooled adolescent sample of repeated cross-sectional surveys.

## Methods

### Description of the Study

An integrated health promotion program was implemented in a high school of Debrecen, Hungary as described elsewhere^1^. Briefly, health education was expanded in the curriculum along with non-compulsory daily classes of physical education (PE); social environment in the school was improved by teaching personality-centered education methods for teachers; and school programs were organized to involve the students' families and the local community, aiming at all students and their parents as well as teachers in the school. A cross-sectional survey among pupils was carried out before the intervention (April 2011, survey 1) that was repeated three times in subsequent years (Fall of 2011: survey 2, Fall of 2012: survey 3; Fall of 2013: survey 4) using identical methods of data collection. All grades from 9 to 12 were included in all survey years.

### Variables

Items of the baseline questionnaire had been taken from the Hungarian version (18) of the Health Behavior of School-aged Children (HBSC) 2010 survey (19).

Life satisfaction (LS) was assessed by the 11-step Cantril-ladder ranging from zero (worst possible life) to 10 (best possible life). Self-rated health (SRH) is a standard single item of HBSC that can be answered on a 4-point scale from excellent to poor that was dichotomized into “good” and “bad” categories in our survey. Body mass index (BMI) was calculated from body weight and body height assessed by the pupils.

Physical activity (PA) was assessed by three questions: (1) the number of times out of school per week during which they were active to the point of sweating, answerable on a 7-point Likert scale ranging from “everyday” to “never”; (2) the number of hours per week out of school during which they were active to the point of sweating, answerable on a 6-point Likert scale ranging from “zero” to “7 or more hours”; (3) the number of days participating in physical activity classes in school (which was available in every school day in this school, as opposed to other schools), with three potential answers (everyday, at least three times, or less than three times per week). A composite variable from all three PA variables was created to assess physical activity with three categories. PA was categorized “vigorous” if the pupil had PA at least four times and 4 h per week in leisure time and attended PA class in school every day; PA was “moderate” if the pupil had PA 2–3 times and 2–3 h in leisure time and attended PA class in school at least 3 days but not every day per week, and pupils were classified “inactive” if they had PA less than two times and <2 h per week in leisure time and attended PA class in school <3 days per week. This method produced a variable of physical activity created only for those students whose responses on all 3 single items were consistent (in bold, 38% of students, Table 1).

**Table 1:** Correlation between variables of physical activity along with number of responses (upper row: correlation coefficient, lower row: significance).

***N***	**Variable**	**1**	**2**	**3**	**4**
3,451	1. Number of weekly activities	1.0000			
3,372	2. Number of active hours per week	−0.8437	1.0000		
		0.0000			
3,446	3. Participation in daily PE class	0.5142	0.5141	1.0000	
		0.0000	0.0000		
1,324	**4. Composite of physical activity**	0.9428	−0.8676	0.5412	1.0000
		0.0000	0.0000	0.0000	

Spearman correlation between the one composite and three single PA variables resulted in significant correlation coefficients (Spearman's rho) reflecting varying correlation as shown in Table 1. Negative correlations appropriately reflected the direction of the given scale from vigorous to inactive.

Demographic data included age assessed by grade (9–12), gender (male/female), and type of permanent place of residence (county seat, city, village, farm), which was dichotomized into “city” and “not city.” Socioeconomic status of the family was assessed by a single item on perceived family wealth (PFW; “How well off do you think your family is?”) answered on a 5-point Likert scale ranging from “not at all well off” to “very well off.” PFW was dichotomized into “well or very well” and “less than well.” This item correlates with a number of health and health behavior outcomes in the HBSC surveys (6, 20).

### Data Collection

A web-based questionnaire was developed for data collection with a standard Linux server using PHP and MySOL support, described in detail elsewhere (see text footnote ^1^). The questionnaire could be completed in 20 min. Access to the questionnaire was pre-organized in a scheduled timepoint for groups of those students in the computer room of the school whose guardians consented to their participation. The test was not available outside of scheduled times. The same questionnaire was used in all four surveys.

### Data Analysis

Data were automatically logged in a database and downloaded to a Microsoft Excel file. After duplicates, empty records and answers out of the specified ranges were removed and data analysis was carried out in STATA 16.1. Continuous variables were compared by *t*-test and categorical variables were analyzed by the chi-square test. Normality test based on skewness and kurtosis was used to test normality of life satisfaction. Though LS is commonly dichotomized (6, 13), we used it as a continuous variable because of problems related dichotomization, the greatest being loss of information (21). Correlation between variables was tested by Spearman's rank order correlation. Association between determinants and outcomes was investigated by simple linear regression, linear regression with the Huber/White sandwich estimator of variance, and heteroskedastic linear regression. The level of significance was set at 0.05.

### Model Selection

Model selection was carried out for several reasons. Life satisfaction was not normally distributed (skewness: −1.51, kurtosis: 7.16), but according to Byrne (22), data can be considered normal if skewness is between −2 and +2 and kurtosis is between −7 and +7. Life satisfaction was heteroskedastic for gender (*p* = 0.022), physical activity (*p* = 0.008), subjective family wealth (*p* < 0.001), grade (*p* < 0.001), and subjective health (*p* < 0.001). Heteroskedasticity was accounted for by carrying out linear regression with the Huber-White sandwich estimator of variance (23), as well as multiplicative heteroskedastic linear regression that models the variance as an exponential function of the variables (24). Another reason for model selection was to compare the appropriateness of the composite PA as opposed to the single item-variables of PA. Each model had life satisfaction as the outcome variable and the same set of independent variables as factor variables (gender, type of permanent residence, subjective family wealth, grade, physical activity, subjective health), as shown in Table 2.

**Table 2:** Model selection for the best model to predict life satisfaction.

	**Life satisfaction**					
**Outcome**	**Model 1**	**Model 2**	**Model 3**	**Model 4**	**Model 5**	**Model 6**
Variable for	Composite	Composite	Composite	Number of	Number of active	Participation in
	of PA	of PA	of PA	weekly activities	hours per week	daily PE class
physical						
activity						
Regression	OLS	OLS w/robust	Hetero-skedastic	OLS w/robust	OLS w/robust	OLS w/robust
		variance estimator	linear regression	variance estimator	variance estimator	variance estimator
Adjusted *R*^2^	0.1587	0.1651	–	0.1509	0.1498	0.1413
Akaike	4,636.927	4,636.927	4,638.927	12,186.44	11,882.01	12,186.76
information						
criterion						

## Results

### Description of the Students

The four surveys took place in one high school in the second largest city of the country producing a large pooled sample (*N* = 3,450). Response rates were 77.67% for the baseline survey, 70.41% for the second, 60.41% for the third, and 64.36% for the fourth survey, calculated from the total number of registered students in each survey year. There was no difference in gender distribution (*p* = 0.607), permanent residence (*p* = 0.682), and perceived family wealth (*p* = 0.276) by survey year, so these variables are described for the entire sample. Girls comprised the majority in all grades in all survey years, and response rates were calculated from the total number of registered students in the survey year (Table 3).

**Table 3:** Description of the students by survey year, grade, and gender.

		**Grade 9**	**Grade 10**	**Grade 11**	**Grade 12**	**Total**	**Response rate (%)**
**Year 1**	Boys (*N*)	102	86	86	55	329	77.67
	Girls (*N*)	120	144	149	110	523	
	Girls %	54.05%	62.61%	63.40%	66.67%	61.38%	
**Year 2**	Boys (*N*)	102	87	73	90	352	70.41
	Girls (*N*)	137	127	115	126	505	
	Girls %	57.32%	59.35%	61.17%	58.33%	58.93%	
**Year 3**	Boys (*N*)	95	44	83	52	274	60.41
	Girls (*N*)	113	60	109	123	405	
	Girls %	54.33%	57.69%	56.77%	70.29%	59.65%	
**Year 4**	Boys (*N*)	95	44	83	52	274	64.36
	Girls (*N*)	113	60	109	123	405	
	Girls %	54.33%	57.69%	56.77%	70.29%	59.65%	

About 77.6% of the pupils were city dwellers, 22.4% lived in villages or farms, with no significant gender difference in permanent residence (*p* = 0.071). Also, 29.7% of the students lived in families perceived to be well or very well off, 63.5% perceived their families as average, whereas 6.8% of them lived in families not so well or not at all well off. This was not different (*p* = b0.199) from those in 9th and 11th grades in the national HBSC sample of 2014 (25).

**Figure 1:**
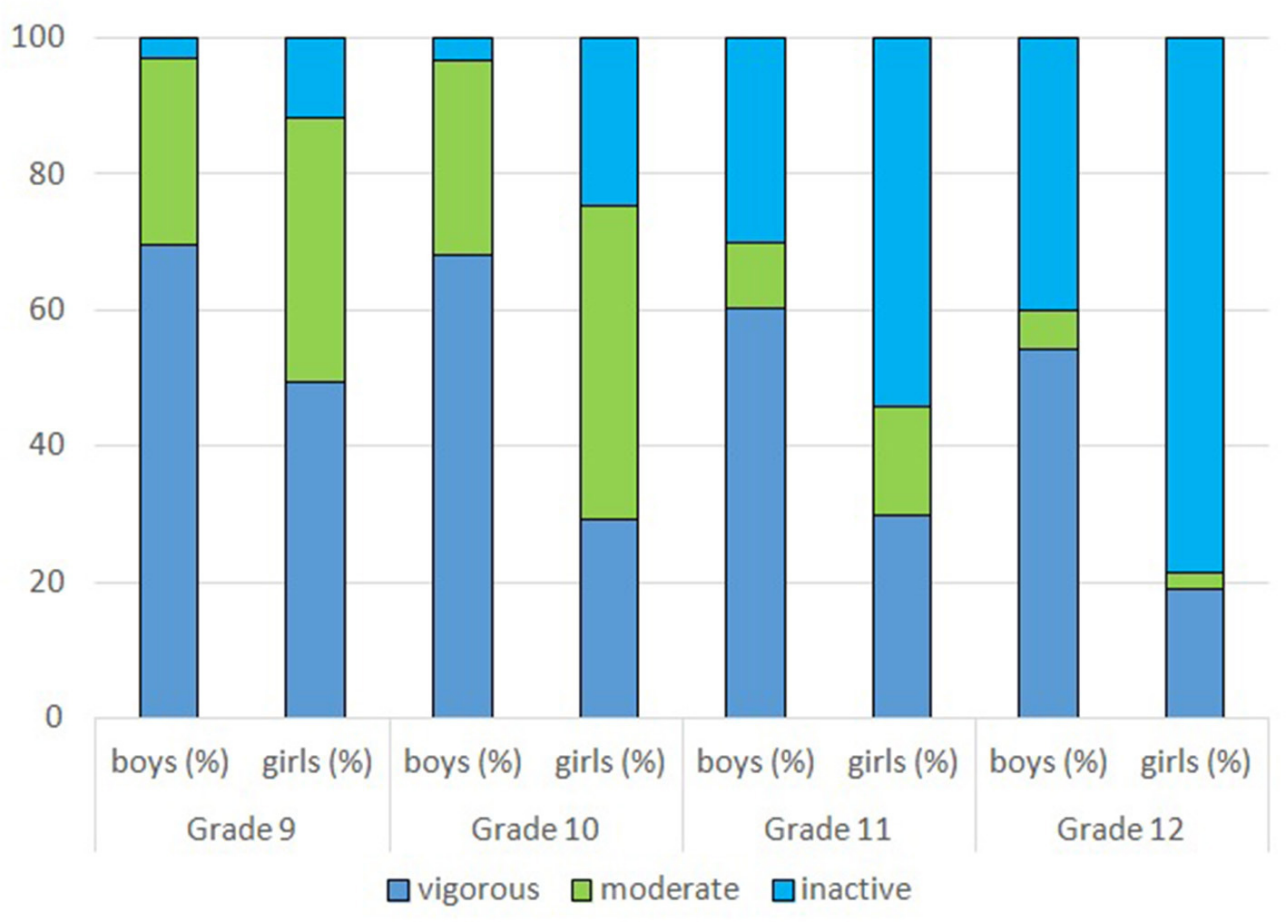
Distribution of physical activity assessed by the composite PA variable by grade and gender.

There was no significant difference by survey year in the distribution of physical activity and life satisfaction (data not shown), but this was not true for grade, so categories of pyhsical activity were analyzed by grade and gender. Physical activity was assessed by three items which were used to classify students into one of the three categories (vigorous, moderate, inactive) of a composite variable as described in Methods Section. Consequently, only those students were classified and shown in Figure 1 who gave consistent answers to all three questions (38% of those who answered all three single-item questions on PA). The proportion of those who were vigorously active per week showed significant gender difference with male advantage in all grades (*p* < 0.001 for all grades), and decreased from Grade 9 to Grade 12 by 15.31% among boys and by 30.43% among girls. Even more dramatic is the rise in the proportion of inactive pupils by 37.2% among boys and by 67% among girls (*p* < 0.001 for both).

Life satisfaction exhibited no significant gender difference (boys: *p* = 0.30; girls: *p* = 0.31). However, it showed a decreasing trend by grade among boys (9th grade: 8.09 ± 1.5; 10th grade :7.98 ± 1.52; 11th grade: 7.88 ± 1.57; 12th grade 12: 7.64 ± 1.73; *p* < 0.001) and girls alike (9th grade: 8.12 ± 1.45; 10th grade: 7.91 ± 1.50; 11th grade: 7.86 ± 1.53; grade 12: 7.85 ± 1.55; *p* = 0.018). There were no significant diffrence between the genders in any grade.

### Correlation of Variables

Life satisfaction, gender, type of permanent residence, perceived family wealth, physical activity, body mass index, self-rated health, and survey years were tested by Spearman correlation (Table 4). Body mass index not being correlated with life satisfaction was omitted, but gender was kept for further analysis.

**Table 4:** Correlation of variables in the surveys (upper row: Spearman's rho, lower row: significance).

	**1**.	**2**.	**3**.	**4**.	**5**.	**6**	**7**.	**8**.	**9**.
1. Life satisfaction	1.0000								
2. Gender	−0.0485	1.0000							
	0.1102								
3. Type of residence	0.1197	−0.0227	1.0000						
	0.0001	0.4551							
4. Perceived family wealth	0.2133	−0.1303	0.1256	1.0000					
	0.0000	0.0000	0.0000						
5. Grade	−0.0924	0.1355	−0.0973	−0.0338	1.0000				
	0.0023	0.0000	0.0013	0.2652					
6. Composite of PA	−0.1539	0.3474	−0.0407	−0.0950	0.3756	1.0000			
	0.0000	0.0000	0.1800	0.0017	0.0000				
7. Body mass index	−0.0279	−0.2469	−0.0204	0.0617	0.1101	−0.0583	1.0000		
	0.3584	0.0000	0.5011	0.0419	0.0003	0.0547			
8. Self-rated health	0.3051	−0.0616	0.0971	0.0676	−0.1179	−0.2192	−0.1123	1.0000	
	0.0000	0.0423	0.0014	0.0258	0.0001	0.0000	0.0002		
9. Date of survey	0.0261	−0.0286	0.0462	0.0328	0.0246	−0.1034	0.0330	0.0287	1.0000
	0.3901	0.3467	0.1279	0.2808	0.4188	0.0006	0.2772	0.3444	

Survey years (date of survey) were not significantly correlated with life satisfaction, but it was further investigated by hierarchical regression defining date as the random effect, and gender, type of residence, perceived family wealth, grade, composite PA, and self-rated health as fixed effects. The hierarchical model was not significantly different from one-level ordinary linear regression (OLS; *p* = 1.000). Next, OLS was carried out including survey dates as independent factor variables of PA, but none of them were significant (survey2: *p* = 0.959, survey3: *p* = 0.553, survey4: *p* = 0.390 compared to survey1), so data from all surveys were pooled for subsequent analysis.

**Figure 2:**
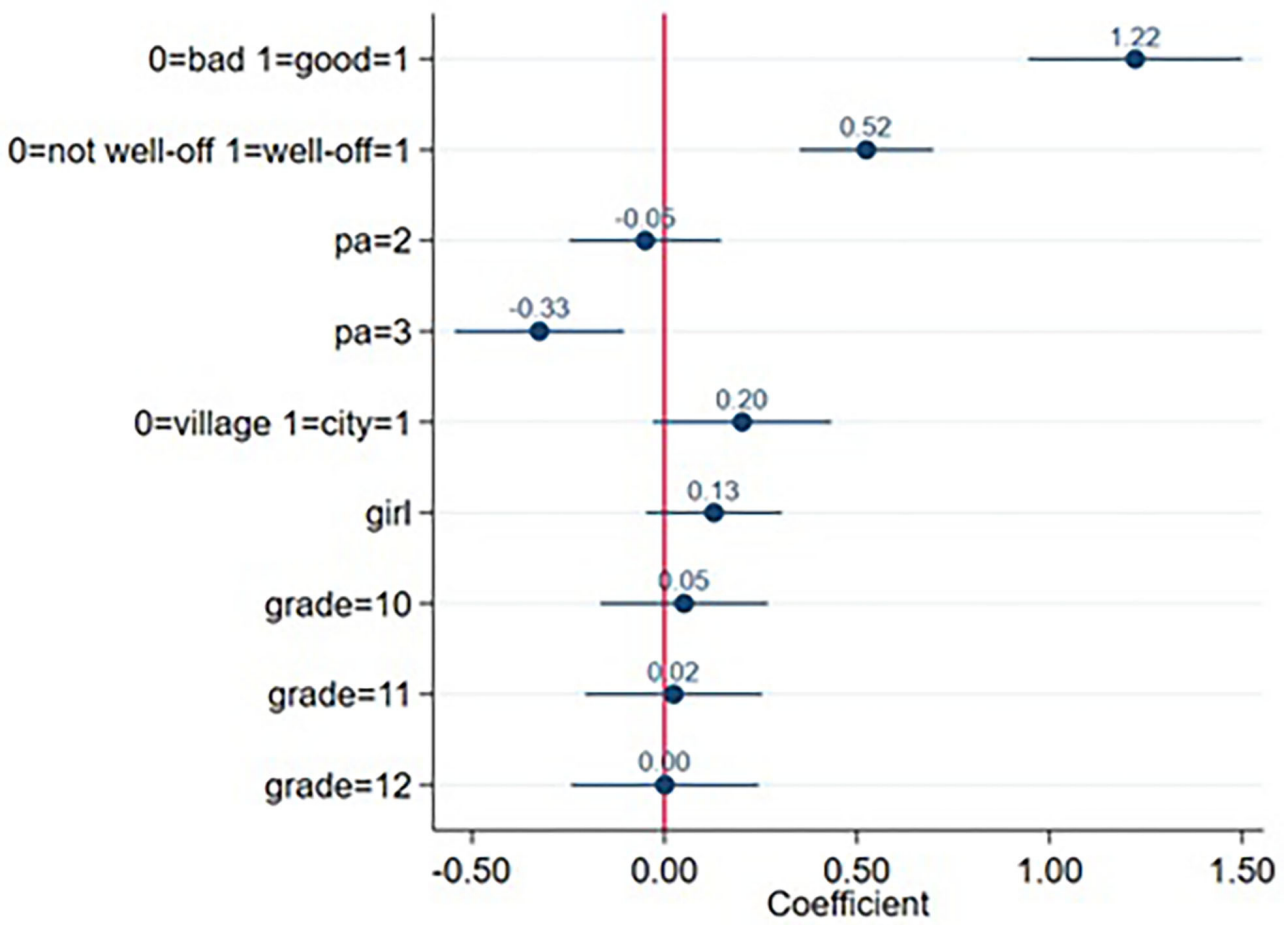
Coefficients and their confidence intervals of independent variables of life satisfaction as outcome in Model 2. Self-rated health: 0: bad 1: good; percieved family wealth: 0:not well-off 1: well-off; physical activity 1:vigorous 2:moderate 3:inactive; permanent place of residence 0:village 1:city;gender: 0:boys 1:girls;grades: 9:9th grade 10:10th grade 11:11th grade 12:12th grade.

Based on the smallest Akaike information criterion and the largest explained proportion of variance, Model 2 was selected to describe determinants of physical activity by OLS with Huber-White sandwich estimator, which is shown in Figure 2 (26). Independent variables are arranged by order of decreasing effect on LS according to the standardized beta coefficients: good self-reported health (as opposed to bad) increased life satisfaction by 0.30 standard deviation; having very well or well-off family (as opposed to not well-off) increased LS by 0.16 standard deviation; and being inactive (pa = 3 in Figure 2) decreased LS by 0.1 standard deviation (as opposed to being vigorously active). Moderate activity (pa = 2) and other independent variables (city as permanent residence compared to village, girls compared to boys, and grades 10, 11, or 12 compared to grade 9) had no significant effect on life satisfaction as shown by the confidence intervals.

## Discussion

Repeated cross-sectional surveys among adolescents of one high school were pooled and analyzed to identify determinants of life satisfaction. The proportion of physically inactive students significantly increased from grade 9 to grade 12, similar to the global trend (27). Self-rated health and living in a well-off family were found to be significant positive determinants, while physical inactivity was found to be a significant negative determinant of life satisfaction in adolescents regardless of gender.

Our findings on the level of physical activity are sadly in concert with that of others. We found a decrease in vigorous physical activity and a worrying increase in the proportion of inactive pupils from grade 9 (15 years old) to grade 12 (18 years old), though the magnitude of change was different by gender. The overall level of physical activity had been low and decreased with age among school-age children in the past two decades according to HBSC data from 32 countries (28). PA also decreased from childhood to adolescence (ages 6–19) across sex in the US National Health and Nutrition Examination Survey (29). A large scale pooled analysis of data from almost 300 studies found that the majority of adolescents around the globe do not get the recommended frequency and intensity of physical activity, and though the prevalence of insufficient physical activity significantly decreased for boys, there had been no change for girls (30).

We identified physical inactivity as having a significant negative effect on life satisfaction with no gender difference. Physical inactivity assessed by various measures was also found among US adolescents to have a negative effect on life satisfaction, but specific for race and gender groups (31). Our study is unusual in the sense that vigorous physical activity was used as the baseline (reference) to which lower levels of PA were compared. This reflects our conviction that vigorous physical activity should be considered the norm among adolescents in line with WHO recommendations (32). However, choosing inactivity as a baseline is more widespread in the literature, so the effects of vigorous activity are more often reported. Data from the 2018 HBSC survey in Lithuania found vigorous PA being a predictor of improved LS in boys but not in girls (13). This study used the same measure of LS as in our surveys, but it was dichotomized and logistic regression was carried out; PA was measured by 2 not clearly differentiating questions, one on frequency and another on duration Another study in Czech and Polish youth (11) applied non-HBSC scales for assessing LS and PA, and used logistic regression to test their association. PA measures were rather cumbersome: transportation and recreation were distinguished, as well as vigorous, moderate activity, and walking that were converted to metabolic equivalent (MET) for tasks without details of the calculation. The study found that girls with the highest life satisfaction reported more PA than girls with the lowest LS, but the results were “not so noticeable” among boys according to the authors. The study is quite difficult to comprehend, but Figure 2 clearly illustrates the large negative effect of physical inactivity on life satisfaction (not mentioned by the authors). A study on Polish lower secondary school students (16) using non-HBSC measures found evidence for physical activity being a predictor of life satisfaction among 13- to 17-year-olds, which is mediated by self-esteem, this being greater among less affluent pupils. Socioeconomic status impacting life satisfaction was also found in our survey similarl to HBSC reports (9).

Advantages of the study include the relatively homogenous sample of students and repeated surveys using identical methods. Since survey dates had no significant impact on life satisfaction, and data could be pooled producing a relatively large sample, which along with the best-selected model counterbalanced potential problems of estimation related to life satisfaction as outcome variable not being normal and unequal error variance in the OLS model. However, standard errors still being somewhat biased cannot be excluded and conclusions should be limited to pupils in similar high schools. Another limitation is the use of the composite PA which resulted in the loss of information since only some one-third of the full sample was used for modeling. However, involving only those who gave consistent answers on physical activity likely increased the reliability of the results. The limitation of the study occurs as all of the analyzed data are from one high school and most of the students are coming from well-off families. All these findings provide even more arguments for the uptake of vigorous activity among adolescents. Vigorous physical activity is not only beneficial for health (10, 33), but also has a positive effect on life satisfaction in part through self-esteem, which is particularly important for those youth who live in less affluent families. Vigorous PA may be a simple and effective tool to reduce health inequalities among adolescents.

## Data Availability Statement

Data are available from the authors at reasonable written request after authorization by the Data Protection Office of the University of Debrecen, Hungary.

## Ethics Statement

Data in the surveys did not include any information that would have enabled personal identification of the students who completed the questionnaires voluntarily and anonymously after informed consent was obtained from their guardians by the school. Only those students were allowed to fill the online questionnaire who had parental consent. Therefore, no formal approval from an ethical review board was required. Written informed consent to participate in this study was provided by the participants' legal guardian/next of kin.

## Author Contributions

KK and ZL-E contibuted to the study design, data collection and analyses, interpretation of results, writing of the manuscript. AS and IV-B took part in the study desing and writing of the original article. All authors contributed to the article and approved the submitted version.

## Funding

KK was supported during the writing of the manuscript by the GINOP-2.3.2-15-2016-00005 project financed by the European Union under the European Social Fund and European Regional Development Fund. Project No. TKP2020-NKA-04 has been implemented with the support provided from the National Research, Development and Innovation Fund of Hungary, financed under the 2020-4.1.1-TKP2020 funding scheme. The funders have had no influence on study design, data collection and analyses, interpretation of results, writing of the manuscript or in the decision to submit it for publication.

## Conflict of Interest

The authors declare that the research was conducted in the absence of any commercial or financial relationships that could be construed as a potential conflict of interest.

## Publisher's Note

All claims expressed in this article are solely those of the authors and do not necessarily represent those of their affiliated organizations, or those of the publisher, the editors and the reviewers. Any product that may be evaluated in this article, or claim that may be made by its manufacturer, is not guaranteed or endorsed by the publisher.

## Footnotes

^1^Erdélyi ZS, Somhegyi A, Balajti I, Kósa K. *Daily optional physical education does not counteract increasing inactivity by age among adolescents*. Manuscript submitted.
